# Supplementary material for: Scutellaria baicalensis Induces Cell Apoptosis and Elicits Mesenchymal–Epithelial Transition to Alleviate Metastatic Hepatocellular Carcinoma via Modulating HSP90β
Source: Int J Mol Sci. 2024 Mar 6;25(5):3073. doi: 10.3390/ijms25053073 (PMC10932101; doi:10.3390/ijms25053073)
Supplement: Supplementary file 1 [file ijms-25-03073-s001.zip › ijms-2854379-supplementary.pdf]

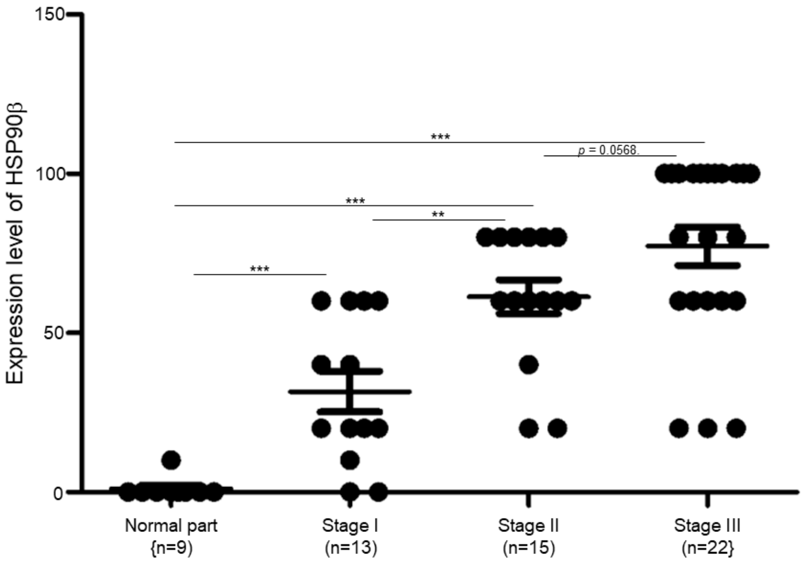

Supplement Figure S1; The quantitative results indicate the different levels of HSP90β in clinical specimens consisting of normal controls (n = 9) and patients with different grades of HCC (n = 50).

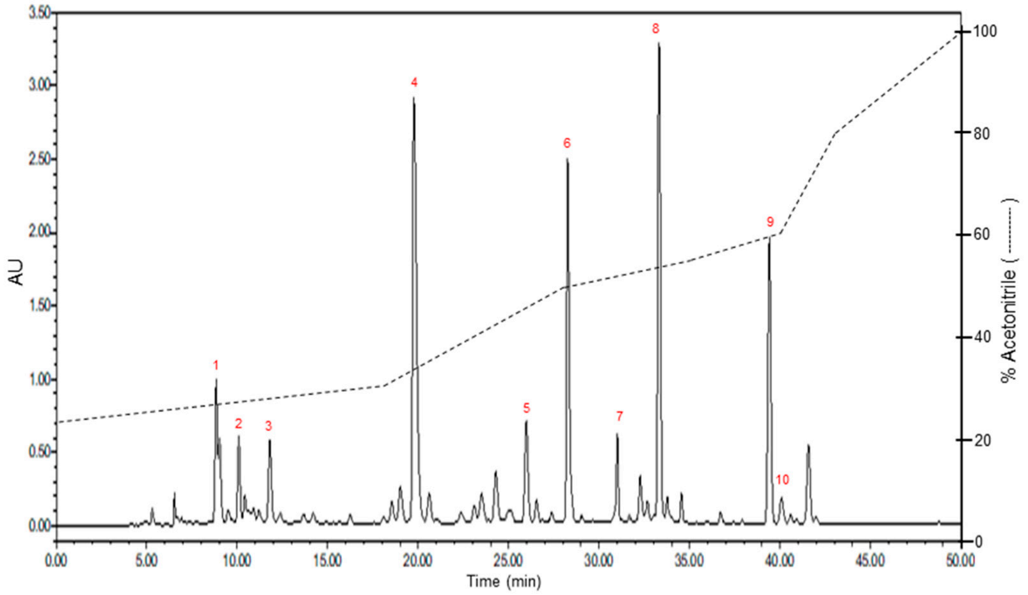

Supplement Figure S2; HPLC-UV$_{277nm}$ chromatograms of ethanol extract derived from *S. baicalensis*. The quantification of samples was determined using a Hitachi L-2130 HPLC system comprising a gradient pump and the Cosmosil 5C18-AR-II, 5 µm (250 ×4.6 mm) column maintained at 35°C. The flow rate was 0.5 mL/min. The major components are including 1:Chrysin-6-C-arabinose-8-C-glucose; 2:Chrysin-6-C-glucose-8-C-arabinose; 3:Baicalin; 4:Galengin-7-O-glucuronide; 5:Chrysin-7-O-β-D-glucuronide; 6:Wogonoside; 7:Norwogonin; 8:Baicalein; 9:Oroxylin A; 10:Chrysin.
